# Characterization of Carbapenem-Resistant Gram-Negative Bacilli Isolates in Multispecialty Private Hospitals in Lagos, Nigeria

**DOI:** 10.3390/idr17050119

**Published:** 2025-09-21

**Authors:** Moruf Salau, Uraiwan Kositanont, Pirom Noisumdaeng, Folasade Ogunsola, Abdul-Wahab Omo-ope Ettu, Damilola Adewojo, Chinonso Ojimma, Omamode Ojomaikre, Kanjana Changkaew

**Affiliations:** 1 Faculty of Public Health, Thammasat University, Pathum Thani 12121, Thailand; moruf.s@fph.tu.ac.th (M.S.); uraiwan.kos@mahidol.ac.th (U.K.); pirom.n@fph.tu.ac.th (P.N.); 2 Thammasat University Research Unit in Modern Microbiology and Public Health Genomics, Pathum Thani 12121, Thailand; 3 Department of Medical Microbiology, University of Lagos, Lagos 101017, Nigeria; fogunsola@unilag.edu.ng; 4 Faculty of Pathology, National Postgraduate Medical College of Nigeria, Lagos 102004, Nigeria; abdul-wahab.ettu@npmcn.edu.ng; 5 Department of Medical Microbiology Laboratory, R-Jolad Hospital, Lagos 100242, Nigeria; damilola.adewojo@rjolad.com; 6 Department of Medical Microbiology Laboratory, Havana Specialist Hospital, Lagos 10001, Nigeria; ojimmanonso2@yahoo.com; 7 Department of General Outpatient, Lagoon Hospitals, Lagos 101000, Nigeria; omamode.ojomaikre@lagoonhospitals.com

**Keywords:** carbapenem-resistant Gram-negative bacilli (CR-GNB), public health, treatment options

## Abstract

Background/Objectives: Carbapenem-resistant Gram-negative bacilli (CR-GNB) pose a growing challenge to public health worldwide due to limited treatment options. This cross-sectional study investigated the characteristics of CR-GNB isolated from clinical specimens in Lagos, Nigeria. Methods: Gram-negative bacilli (GNB) and clinical data were obtained from three multi-specialist private hospitals between March and June 2023. The GNB were identified using the Analytical Profile Index (API) and investigated for CR-GNB by disk diffusion. Antimicrobial resistance patterns and carbapenemase gene data for presumptive carbapenemase-producing Gram-negative bacilli (CP-GNB) were analyzed using Vitek-2 and polymerase chain reaction (PCR). Results: Of 317 GNB, 29.0% (n = 92) were CR-GNB. Significantly higher numbers of CR-GNB were reported from the intensive care unit and oncology department (*p* = 0.009). Of all CR-GNB, 17 isolates (18.5%) were classified as presumptive CP-GNB. In this subgroup, resistance rates of ampicillin/sulbactam (100.0%) and trimethoprim/sulfamethoxazole (100.0%) were highest. Ten (10) CP-GNB were confirmed, representing 3.15% of all GNB tested. Seven isolates of New Delhi Metallo-β-lactamase (*bla*_NDM_) were found among *P. aeruginosa*, *K. pneumoniae*, *E. coli*, and *A. baumannii*. The *bla*_NDM_ was identified in strains classified as extensively drug-resistant (XDR) and pandrug-resistant. Conversely, the *bla*_KPC_ was detected solely in multidrug-resistant and XDR strains. Conclusions: Emerging CR-GNB, specifically CP-GNB, in Nigeria emphasize the need for specific therapeutic management of infected patients. Antimicrobial stewardship and long-term surveillance efforts must be implemented in healthcare settings, as well as improved, accelerated microorganism identification techniques.

## 1. Introduction

Antimicrobial resistance (AMR) poses a growing challenge to global health. Recognizing the escalating nature of this threat, the World Health Organization (WHO) designated a specific bacterial priority pathogens list (BPPL) to guide research and facilitate novel drug development [1]. These pathogens include carbapenem-resistant Gram-negative bacilli (CR-GNB), such as *Acinetobacter* and *Pseudomonas*, and Enterobacterales. Since these pathogens can produce carbapenemase that render beta-lactam antimicrobials ineffective, they have the potential to spread widely and cause outbreaks with unique variabilities in prevalence and characteristics in diverse settings [1].

CR-GNB develop antimicrobial resistance through various genetic and biochemical adaptations. The widespread emergence of CR-GNB is largely driven by carbapenemase production, a resistance mechanism propagated through the horizontal transfer of encoding genes. Owing to their capacity to hydrolyze various β-lactam antimicrobials, including carbapenems, cephalosporins, penicillin, and aztreonam, Carbapenemase-producing Gram-negative bacilli (CP-GNB) are clinically important. They pose a significant threat by compromising the activity of the last-resort antimicrobials used for treating severe infections. Moreover, epidemiologically, carbapenemase genes can disseminate into various bacteria across almost all geographic regions [2]. CP-GNB are more virulent and associated with higher levels of AMR, worse health outcomes, and more rapid spread.

The variation in CP-GNB’s prevalence is influenced by carbapenemase production. which is categorized into different Ambler classes. Classes A, C, and D carbapenemase include the β-lactamases with serine at their active site, whereas the molecular class B carbapenemase includes metalloenzymes with a zinc active site [2]. Ambler classes A, B, and D carbapenemase are clinically significant among nosocomial pathogens. Class A is notably represented by the *Klebsiella pneumoniae* carbapenemase (KPC), class B includes MBLs such as New Delhi Metallo-β-lactamases (NDM), Verona integron-encoded (VIM), and imipenemase (IMP) enzymes, and class D comprises carbapenem-hydrolyzing oxacillinases like OXA-48 [2]. Class D includes carbapenem-hydrolyzing oxacillinase (OXA), such as the OXA-48 enzymes [3,4]. Carbapenemase genes were initially found to be chromosomally mediated in specific bacterial species. However, they can now also be mediated by plasmids. This horizontal transfer facilitates the faster spread of resistance between different bacterial species and genera [5]. The most frequently reported plasmid-mediated carbapenemase genes include *bla*_VIM_, *bla*_IMP_, *bla*_KPC_, *bla*_NDM_ and *bla*_OXA-48_, which vary across continents [6,7].

In Nigeria, studies in public hospitals have reported a high prevalences of CR-GNB. The most prevalent CR-GNB species are *Escherichia coli*, *Klebsiella pneumoniae*, and *Pseudomonas aeruginosa* [8,9]. A review of these studies revealed a national prevalence average of 21.3%, with *bla*_NDM_ and *bla*_VIM_ as the most detected genes [8]. It must be noted that most of the studies focused on Enterobacterales using regional data that varied according to each study. For example, a 7.7% prevalence of carebapenem-resistant Enterobacterales was reported among hospitalized patients in South-west Nigeria, while a 29% prevalence was reported in Southeast Nigeria [10,11]. A more comprehensive study that profiled CR-GNB reported a prevalence of 22% for CR-GNB in a tertiary hospital in South-west Nigeria [9].

Infections caused by carbapenem-resistant microorganisms have worse clinical outcomes than those caused by carbapenem-sensitive microorganisms [12,13]. Some of the notable risk factors for increased mortality are past antimicrobial usage, prolonged hospital stays, surgical interventions, the presence of indwelling devices, intensive care unit (ICU) admission, and extended hospitalization [14,15]. The studies conducted in Nigerian public hospitals over the past ten years indicate that the threat of CR-GNB is escalating [4,6,16,17]. However, despite this and also the vital role of private hospitals in patient care in Nigeria—particularly in Lagos State, which has a population of over 24 million and a private hospital sector that serves more than 60% of its residents—there is sparse data on CR-GNB in these settings [18]. This study thus aimed to investigate the characteristics of CR-GNB, including the occurrence of CP-GNB isolated from clinical specimens in Lagos, Nigeria. This comprehensive data, encompassing both phenotype and genotype, is imperative for the effective treatment and enhanced control of AMR.

## 2. Materials and Methods

### 2.1. Study Design and Setting

A cross-sectional study was conducted from March to June 2023 at three multispecialty private hospitals in Lagos, Nigeria: R-Jolad Hospital, Lagoon Hospitals, and Havana Specialist Hospital. These facilities span the three senatorial districts and collectively serve a diverse patient population. R-Jolad Hospital has 215 beds, Lagoon Hospitals has a total of 120 beds across its branches, and Havana Specialist Hospital has 100 beds. While none of these hospitals have a formal antimicrobial stewardship program, they do implement infection prevention and control initiatives.

These hospitals were meticulously chosen for their exceptional ability to manage a diverse range of medical cases and their proficiency in conducting in-house laboratory testing. Furthermore, functional electronic medical records that connect laboratories to other hospital units were important selection criteria. In total, 317 Gram-negative bacilli (GNB) were collected from both inpatients and outpatients, without a specific focus on individuals with severe infections or hospitalizations. These samples were derived from routine laboratory microbiological investigations and were subsequently tested for carbapenem resistance in the next stage of analysis. Among the isolates, based on the presence of clinical signs and symptoms indicative of infection, 296 were identified as responsible for clinical infections, and 21 were categorized as colonizers. Moreover, to identify the associated factors, the patients’ relevant clinical records were obtained after their discharge or death.

### 2.2. Identification of Gram-Negative Bacilli

Consecutive, non-duplicate isolates of GNB were obtained from the chosen hospitals. The bacterial isolates were recovered from a variety of clinical samples, including urine, high vaginal swab, stool, wound swab, ear swab, throat swab, sputum, catheter tips, semen, tracheal aspirate, and blood. These isolates were cultured on MacConkey agar (Oxoid, Thermo Fisher Scientific, London, UK) to differentiate between lactose fermenters and non-lactose fermenters. From each agar plate, distinct colonies were selected and cultured on Mueller-Hinton agar (Oxoid, Thermo Fisher Scientific, London, UK). Unique identification numbers were assigned to each isolate in mixed cultures to manage potential errors. The oxidase test was then performed using MAST ID™ oxidase strips (MAST Group Limited, Merseyside, UK). All the isolates were then subjected to Gram staining (Sigma-Aldrich, Burlington, MA, USA), and quality control was maintained using *P. aeruginosa* ATCC 27853 and *E. coli* ATCC 25922. Gram staining was performed according to the standard protocol established by the American Society for Microbiology [19]. Finally, the pure isolates were identified using API 20 NE for oxidase-positive non-lactose fermenters and API 20E for oxidase-negative lactose fermenters by following the manufacturer’s instructions (bioMérieux, Marcy l’Etoile, France). To minimize misidentification, colony morphology, Gram staining, oxidase testing, and quality control strains (*P. aeruginosa* ATCC 27853 and *E. coli* ATCC 25922) were used for cross-validation (Table S1).

### 2.3. Detection of Carbapenem-Resistant Gram-Negative Bacilli

The identified GNB were tested for CR-GNB by checking their antimicrobial susceptibility to ertapenem, imipenem, and meropenem (MAST Group Ltd., Merseyside, UK). The standard and interpretive criteria recommended by the European Committee on Antimicrobial Susceptibility Testing (EUCAST) guidelines were followed [20]. In our study, isolates classified as ‘category I’ (now termed ‘susceptible, increased exposure’) were combined with other susceptible isolates under the non-resistant group, in accordance with local laboratory reporting protocols. Subsequently, the isolates demonstrating potential resistance to ertapenem, imipenem, or meropenem were further screened for CP-GNB using a minimum inhibitory concentration (MIC) test via the Vitek 2 system (bioMérieux, Marcy l’Etoile, France). This test was interpreted using the Clinical and Laboratory Standards Institute (CLSI) guidelines [21], and based on the test results, the isolates that showed potential resistance or intermediate susceptibility to imipenem or meropenem were categorized as presumptive CP-GNB [22,23]. In this study, a combined methodology was employed, involving disk diffusion testing followed by EUCAST guidelines [20] and MIC testing, which applied CLSI interpretive criteria [21]. Although this dual approach may appear inconsistent, it reflects the standard workflow in the participating laboratories.

### 2.4. Antibacterial Susceptibility Test of the Presumptive Carbapenemase-Producing Gram-Negative Bacilli

The CP-GNB were tested for antimicrobial susceptibility to 14 antimicrobial agents using Vitek 2 AST cards (bioMérieux, Marcy l’Etoile, France). The antimicrobials tested were ampicillin/sulbactam, trimethoprim/sulfamethoxazole, piperacillin/tazobactam, cefotaxime, ceftazidime, ceftazidime/avibactam, ceftolozane/tazobactam, cefepime, imipenem, meropenem, gentamicin, ciprofloxacin, amikacin, and colistin. The MIC and clinical breakpoints were determined and interpreted using the CLSI guidelines [21]. Quality control was performed using *P. aeruginosa* ATCC 27853 and *E. coli* ATCC 25922. The antimicrobial resistance pattern was divided into three levels. Multidrug-resistant (MDR) organisms were defined as microorganisms that demonstrate non-susceptibility to ≥1 antimicrobial agent in ≥3 antimicrobial categories. Extensively drug-resistant (XDR) organisms were defined as “non-susceptible to at least one agent in all but two or fewer antimicrobial categories”. Pandrug-resistant (PDR) organisms were defined as non-susceptible to all antimicrobial agents in all antimicrobial categories [24].

### 2.5. Molecular Detection of Carbapenemase Genes

The identified CR-GNB’s DNA templates were prepared using the commercial DNA extraction kit (NIMR, Lagos, Nigeria). PCR techniques were employed to detect five significant carbapenemase genes. The PCR mixture (total 20 µL) comprised 1X FIREPol Master Mix (Solis Biodyne, Tartu, Estonia), 0.2 µM of each primer shown in Table 1, 2 µL of DNA template (10–100 ng), and deionized water (which was used to make the reaction mixture). Amplification was conducted in a Techne Prime thermal cycler (Cole Palmer, Vernon Hills, IL, USA) with the PCR conditions shown in Table 1. The PCR products were analyzed using electrophoresis on a 1.5% agarose gel with SYBR Safe stain (Thermo Fisher Scientific, Waltham, MA, USA), and their sizes were determined based on a 100 bp DNA ladder (Solis Biodyne, Tartu, Estonia). After electrophoresis, the DNA bands were visualized under a UV transilluminator (Biobase, Jinan, China). The positive controls for *bla*_KPC_, *bla*_VIM_, *bla*_NDM_, *bla*_IMP_, and *bla*_OXA-48_ detection were *K. pneumoniae* NCTC 13438, *K. pneumoniae* NCTC 13440, *K. pneumoniae* NCTC 13443, *E. coli* NCTC 13476, and *K. pneumoniae* NCTC 13442, respectively (Table S1).

### 2.6. Data Analysis

In a previous study conducted in Nigeria, the prevalence of CR-GNB was 22% (39/177 isolates) [9]. Based on this, we estimated a minimum sample size of 326 isolates using a one-proportion formula at a 95% confidence level and a 4.5% margin of error.

AMR was analyzed using the WHONET 2023 software version. Statistical analyses were performed using the SPSS program 18 version for Windows (PASW serial no. 5082357) (SPSS Inc., Chicago, IL, USA). The data were analyzed using descriptive statistics (e.g., frequency distribution, percentage, and interquartile range). Pearson’s chi-square and Fisher’s exact tests were used to analyze the statistical differences in the proportions of non-carbapenem-resistant and carbapenem-resistant isolates based on their expected values. Depending on the data distribution, an independent *t*-test was conducted to compare the number of non-carbapenem-resistant and carbapenem-resistant isolates within the interquartile range. A confidence interval of 95% was established, with a *p*-value of less than 0.05 required for statistical significance.

## 3. Results

### 3.1. The Identified Gram-Negative Bacilli Isolate

**Figure 1:**
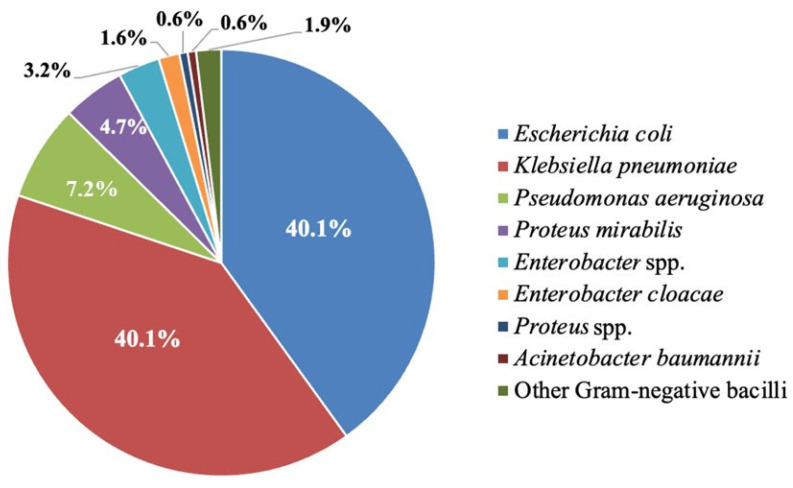
The proportion of identified Gram-negative bacilli in this study (N = 317 isolates).

In total, 317 consecutive non-duplicate isolates of GNB were obtained. Of these, 261 (82.3%) GNB isolates were obtained from patients who presented to the general outpatient departments in all the chosen hospitals. The identified bacteria corresponded to *Escherichia coli* (40.1%) and *Klebsiella pneumoniae* (40.1%), accounting for about 80.1% of the isolates. In addition, *Pseudomonas aeruginosa* (7.2%), *Proteus mirabilis* (4.7%), *Enterobacter* species (3.2%), and *Enterobacter cloacae* (1.6%) were also prominent, collectively representing 16.7% of the isolates (Figure 1).

### 3.2. Demographic Distribution of Carbapenem-Resistant Gram-Negative Bacilli

Table 2 presents the patients’ demographic characteristics, the origins of the specimens, and the prevalence of CR-GNB isolates. Among the analyzed GNB, 92 isolates (29.0%) were identified as CR-GNB based on susceptibility testing to ertapenem, imipenem, and meropenem. The median age of patients with CR-GNB isolates (38.5 years, IQR 29.8–49.0) was slightly higher than that of patients with non-CR-GNB isolates (36 years, IQR 29–45), with the difference reaching borderline statistical significance (*p* = 0.050). Notably, there was no significant difference in the prevalence of CR-GNB isolates between male and female patients. Although most of the isolates were collected from outpatients, there was no statistically significant difference between the outpatient and inpatient settings (*p* = 0.196). Moreover, a significant distinction was found between the general medicine departments in the outpatient setting, where CR-GNB isolates were identified less frequently compared to non-CR-GNB isolates (*p* < 0.001). In contrast, CR-GNB isolates were more prevalent (3.8%) in the oncology outpatient departments, whereas none were detected in the non-resistant group (*p* = 0.028). Within inpatient settings, a significantly higher proportion of CR-GNB isolates were recovered from patients admitted to ICUs (*p* = 0.001). Among the analyzed specimen types, CR-GNB isolates were most frequently found in urine samples (79.3%), followed by high vaginal swabs (6.5%) and ear swabs (4.3%). However, we did not find any significant differences in the frequency of CR-GNB isolates across the different specimen types.

### 3.3. Prevalence and Proportion of Carbapenem-Resistant Gram-Negative Bacilli

Out of the 317 Gram-negative bacilli isolates examined, 92 (29.0%) were identified as resistant to carbapenems. The most prevalent carbapenem-resistant organisms were *E. coli* (46.7%) and *K. pneumoniae* (28.3%), followed *by P. aeruginosa* (14.1%). Other species exhibiting carbapenem resistance included *P. mirabilis* (6.5%), *A. baumannii* (2.2%), *E. asburiae* (1.1%), and *E. fergusonii* (1.1%). Moreover, several species were exclusively present in the non-carbapenem-resistant group and were not detected among the resistant isolates. These species included *E. cloacae*, *A. fergusonii*, *B. cepacia*, *R. ornithinolytica*, and *Salmonella enterica* (Table 3).

### 3.4. Antimicrobial Resistance Profiles and Carbapenemase-Encoding Genes of Presumptive Carbapenemase-Producing Gram-Negative Bacilli

The MIC screening of CR-GNB using imipenem and meropenem identified 17 out of 92 isolates (18.5%) as presumptive CP-GNB. Of the 17 CP-GNB, the resistance rates of ampicillin/sulbactam (100.0%) and trimethoprim/sulfamethoxazole (100.0%) were the highest, followed by piperacillin/tazobactam (94.1%), cefotaxime (94.1%), and ceftolozane/tazobactam (88.2%). In contrast, the resistance rate of colistin (11.8%) was the lowest. The resistance rates of imipenem and meropenem were 82.4% and 58.8%, respectively. Most of the presumptive CP-GNB (11 isolates) were XDR, demonstrating non-susceptibility to at least one antimicrobial agent in all but a maximum of two antimicrobial categories (Table 4).

Out of the presumptive 17 CP-GNB, 10 (58.8%) were carbapenemase-producing strains. Overall, the 10 CP-GNB accounted for 3.15% of the GNB. Specifically, the genes *bla*_NDM_ and *bla*_KPC_ were detected in seven and three isolates, respectively (Figures S1 and S2). We could not identify the presence of the *bla*_VIM_, *bla*_IMP_, and *bla*_OXA_. The *bla*_NDM_ was found in *P. aeruginosa*, *K. pneumoniae*, *E. coli*, and *A. baumannii*. However, the *bla*_KPC_ was exclusively associated with *K. pneumoniae*. The *bla*_NDM_ was identified in the strains classified as XDR (six isolates) and PDR (one isolate). Conversely, the *bla*_KPC_ was detected solely in MDR (two isolates) and XDR (one isolate) (Table 4).

### 3.5. Demographic Data and Clinical Outcome of Presumptive Carbapenemase-Producing Gram-Negative Bacilli

As Table 5 shows, the presumptive CP-GNB was predominantly found in general medicine/surgery outpatient departments, primarily from the urine specimens collected from patients exhibiting signs of presumptive urinary tract infections. Most of the patients were subsequently discharged without any recorded mortality within 30 days. Regrettably, a patient (L19) in the ICU succumbed to a benign neoplasm, specifically a pituitary adenoma, with carbapenem-resistant *E. coli* identified in the blood sample. This strain also exhibited PDR, indicating resistance to all antimicrobial agents across the tested categories, including colistin, the last-resort antimicrobial.

## 4. Discussion

AMR remains a major public health challenge, particularly in low-resource regions such as Western Sub-Saharan Africa, including Nigeria. Limited data on AMR from these settings underscore the need to strengthen microbiology laboratory capacity and surveillance systems [29]. This study addressed this gap by assessing the prevalence of CR-GNB in multispecialty private hospitals—a sector where AMR data is scarce. Our study found that 29% of GNB isolates were CR-GNB, higher than the previously reported national mean of 20.5% in Nigeria [8]. This rise may be linked to the nature of multispecialty private hospitals, where antimicrobial prescribing is often less regulated and complicated infections are commonly treated. The significant differences in CR-GNB prevalence across Nigeria highlight how factors such as patient selection, hospital type, and laboratory methods affect resistance estimates, underscoring the need for improved surveillance in private facilities.

Our study highlights notable differences in the distribution of CR-GNB versus non-CR-GNB isolates across clinical settings. Although the overall outpatient proportion was not statistically different (*p* = 0.196), the CR-GNB isolates were significantly less common in general medicine departments in the outpatient setting, suggesting a lower risk among outpatients with less severe conditions. This finding aligns with previous studies, which found higher rates of multidrug-resistant organisms in inpatient and critical care settings, driven by increased antimicrobial exposure and the use of invasive procedures [30]. Consistently, a significantly higher prevalence of CR-GNB was observed in ICUs and oncology departments (*p* = 0.009), where patients are more vulnerable due to immunosuppression, prolonged antimicrobial use, and device-associated care [31,32]. The increased use of carbapenems—often as last-resort agents—further contributes to selective pressure in these high-risk units [32]. Notably, 82.3% of the isolates belonged to outpatients, which limited inpatient representation. The small number of inpatients may lead to an underestimation of CR-GNB burden in hospitalized populations. Future studies should thus ensure a more balanced sampling to better reflect AMR in inpatients, particularly in Nigeria, where surveillance of CR-GNB remains limited.

In our data, patients with CR-GNB (38.5 years, IQR 29.8–49.0) were slightly older than those non-CR-GNB (36 years, IQR 29–45), although most were still young adults. This profile is modestly younger than a hospital-based cohort from South-west Nigeria (median 42 years, IQR 29–64), in which the highest CRE proportion occurred among adults aged 40–54 years [9]. By contrast, consolidated national surveillance of CRE bloodstream infections (BSI) in South Africa described a younger case mix overall (median age ~31 years) [33]. Finally, a recent ICU study from Kenya did not report a median age but found that nearly half of ICU patients with GNB infections were between 40 and 60 years old, consistent with an older inpatient case mix typical of critical care [34]. Taken together, our median age lies between Nigerian inpatient cohorts and South African CRE BSI surveillance, and the differences likely reflect setting (outpatient vs. inpatient/ICU), case mix, and study focus (all GNB vs. CRE from bloodstream infections). Careful comparison across studies should therefore account for these design and population differences.

Consistent with reports from Nigeria, the predominant CR-GNB in our study were *Escherichia coli* and *Klebsiella pneumoniae* [8]. Our observed resistance rates were higher than those reported in several West African countries but lower than rates from Southern Africa and Egypt [35]. The differences observed may be attributed to variations in study design, patient selection, hospital type, and laboratory methods. Additionally, these variations could be influenced by local healthcare practices, including antimicrobial prescribing behaviors, referral patterns, and the implementation of stewardship programs. Further research is necessary to clarify how these contextual factors shape regional resistance patterns.

Using carbapenemase gene detection, we identified 10 CP-GNB, representing 3.15% of all the GNB—higher than the 2.7% previously reported in Nigeria [36]. These isolates exhibited broad resistance across antimicrobial classes, underlining the role of carbapenemases in AMR [4,16,37]. Moreover, we detected MDR, XDR, and PDR phenotypes in the identified CP-GNB, thereby posing substantial challenges for clinical management and AMR surveillance [4,29]. Notably, CRE, CRAB, and CRPA exhibited resistance to last-line agents, such as piperacillin/tazobactam, ceftolozane/tazobactam, ceftazidime/avibactam, and cefepime. Although these antimicrobials typically demonstrate high efficacy against GNB—with susceptibilities up to 99% among Enterobacterales [38,39]—their effectiveness may be limited in difficult-to-treat infections. For instance, susceptibility among difficult-to-treat *P. aeruginosa* was reported to be under 20% [40]. Despite not specifically assessing such infections, our observed high resistance rates raise concerns.

Among CP-GNB, the predominant carbapenemase genes were *bla*_NDM_ and *bla*_KPC_, differing slightly from previous Nigerian data that highlighted *bla*_NDM_ and *bla*_VIM_ [8]. The *bla*_NDM_ genes, particularly *bla*_NDM-1_, are plasmid-mediated and highly adaptable, indicating widespread horizontal transmission among bacteria. Additionally, the absence of a standardized test for metallo-beta-lactamases leads to many unrecognized asymptomatic carriers [41]. A recent study in Lagos found that plasmids harboring carbapenemase genes shared structural similarities with those from Asia, Australia, and Europe, suggesting active global transmission [4]. While *bla*_KPC_ was detected exclusively in *K. pneumoniae*, it remains uncommon in Nigeria and was first reported in 2015 [42]. The gene’s association with plasmids enhances its epidemic potential and limits its treatment options [43].

Despite *A. baumannii*’s clinical relevance, Nigerian healthcare settings are limited in their capacity to detect it [15]. *A. baumannii* often exhibit high resistance to essential antimicrobials [17], with a recent prevalence of Carbapenem-resistant *A. baumannii* (CRAB) carrying the *bla*_NDM_ gene at 27.9% [44]. We identified two *A. baumannii* from one of the hospitals, albeit smaller in other studies. However, these recovered *A. baumannii* were *bla*_NDM_-carrying CRAB, indicating a 100% prevalence among these organisms in our sample. Our findings align with the propensity of *A. baumannii* to develop XDR. These two organisms were susceptible to amikacin and colistin, which is consistent with the findings of a study in public tertiary hospitals in Nigeria [17]. Moreover, our findings support the recommendation of treating CRAB with a combination of amikacin and colistin or minocycline with cefoperazone-sulbactam [31]. A study in China suggests that a combination of amikacin, polymyxin B, and sulbactam can effectively combat MDR *A. baumannii* [45].

Although *A. baumannii* was susceptible to amikacin and colistin, our study showed that *A. baumannii* still retains the potential to disseminate *bla*_NDM_ within healthcare settings and environments, leading to potential outbreaks [4,46].

Only one death was reported for the 30-day all-cause mortality outcome among patients with infections caused by CP-GNB in this study. The organism isolated from the patient’s sample was a PDR *E. coli*, which caused bloodstream infection. The patient had undergone transsphenoidal surgery for benign neoplasia and was admitted to the ICU. Although the patient’s underlying condition and surgical complications may have contributed to the fatal outcome, the presence of a PDR bloodstream infection likely exacerbated the clinical course, consistent with previous reports on the high risk associated with bacteremia [47]. Furthermore, the mortality observed in this study was lower than the rates reported in larger cohorts from Africa, which are approximately 37.2% [15]. This discrepancy is likely due to the small sample size and the predominance of outpatient cases among our isolates. As a result, the actual mortality burden of CR-GNB in hospitalized patients may be underestimated in our findings. Nevertheless, this fatal case illustrates the potential severity of CP-GNB infections and underscores the importance of early detection, infection prevention, and antimicrobial stewardship interventions.

Our study has limitations, particularly in the context of antimicrobial susceptibility testing. Species identification in this study relied on API 20E and API 20NE kits, supplemented by morphology, Gram staining, oxidase testing, and quality control strains. Although widely used in clinical laboratories, these methods have limited accuracy, particularly for distinguishing *Enterobacter* and *Acinetobacter* [48,49]. The lack of molecular confirmation methods, such as 16S rRNA sequencing or MALDI-TOF mass spectrometry, is a notable limitation of this study. Due to resource constraints, these methods could not be utilized. MALDI-TOF mass spectrometry and 16S rRNA sequencing provide greater reliability: the former achieved 100% concordance in identifying *Acinetobacter* with an updated database [50,51], while both methods demonstrate superior speed and precision in routine identification of *Enterobacter* [52]. We employed the EUCAST method to test carbapenem resistance via agar disk diffusion and the CLSI method to determine the minimum inhibitory concentrations of presumptive CP-GNB. Changes to the standard guidelines may thus impact our AMR assessments. Furthermore, classifying isolates with intermediate inhibition zones as non-resistant may underestimate the actual resistance rates. Another key limitation is that we relied solely on resistance to imipenem or meropenem to identify potential carbapenemase-producing organisms—we might have thus missed cases of low-level resistance. This is due to the variable expression of the carbapenemase gene, porin mutations, or efflux mechanisms [53]. Finally, the short study duration may have underestimated the prevalence of CR-GNB. The achieved sample size (317 vs. 326) was <3% below the target, increasing the margin of error only marginally (4.5% to 4.6%). Such a minor shortfall is unlikely to affect the validity of the findings and is generally acceptable in biomedical research when acknowledged as a limitation [54].

## 5. Conclusions

Our study offers key insights into the characterization of CR-GNB and CP-GNB in multispecialty private hospitals in Lagos, Nigeria. *P. aeruginosa* and *E. coli* were found to be the predominant carbapenem-resistant species, with a high prevalence of extended drug-resistant strains among CP-GNB. Moreover, the frequent detection of *bla*_NDM_ along with *bla*_KPC_ underscores the need for tailored therapeutic strategies. Accurate detection—beginning with screening for ertapenem, meropenem, or imipenem resistance and confirmed by phenotypic and genotypic methods—is essential for timely treatment and infection control. Given the evolving nature of AMR in healthcare settings, continuous surveillance, antimicrobial stewardship, and robust infection prevention measures are imperative. Our findings support the urgent need for government-led interventions that target the private healthcare sector as part of Nigeria’s broader AMR response.

## Figures and Tables

**Table 1:** Oligonucleotide primers and conditions used in this study.

Primer	Sequence (5′–3′)	Amplicon Size (bp)	PCR Condition (35 Cycles)			Reference
			Denature	Annealing	Extension	
*Bla*_NDM_-F	GGGCAGTCGCTTCCAACGGT	475	95 °C	58 °C	72 °C	[25]
*Bla*_NDM_-R	GTAGTGCTCAGTGTCGGCAT		30 s	30 s	1.30 s	
*Bla*_OXA_-F	TTGGTGGCATCGATTATCGG	438	95 °C	55 °C	72 °C	[26]
*Bla*_OXA_-R	GAGCACTTCTTTTGTGATGGC		30 s	30 s	1.30 s	
*Bla*_KPC_-F	CATTCAAGGGCTTTCTTGCTGC	538	95 °C	55 °C	72 °C	[27]
*Bla*_KPC_-R	ACGACGGCATAGTCATTTGC		30 s	30 s	1.30 s	
*Bla*_IMP_-F	GGAATAGAGTGGCTTAAYTC	232	95 °C	55 °C	72 °C	[28]
*Bla*_IMP_-R	TCGGTTTAAYAAAACAACCACC		30 s	30 s	1.30 s	
*Bla*_VIM_-F	GATGGTGTTTGGTCGCATA	390	95 °C	55 °C	72 °C	[28]
*Bla*_VIM_-R	CGAATGCGCAGCACCAG		30 s	30 s	1.30 s	

Note: *bla*_NDM_—New Delhi Metallo-β-lactamase; *bla*_OXA_—Oxacillinase-type β-lactamase; *bla*_KPC_—*Klebsiella pneumoniae* carbapenemase; *bla*_IMP_—Imipenemase Metallo-β-lactamase; *bla*_VIM_—Verona Integron-encoded Metallo-β-lactamase; F—Forward Primer; R—Reverse Primer.

**Table 2:** Prevalence of non-carbapenem-resistant Gram-negative bacilli (non-CR-GNB) and carbapenem-resistant Gram-negative bacilli (CR-GNB) by demographics, setting, and specimen type.

	Non-CR-GNB (n = 225)	CR-GNB (n = 92)	*p*-Value
**Age, year (IQR)**	36 (29–45)	38.5 (29.8–49.0)	0.050 ^c^
**Sex, n (%)**			0.758 ^a^
Female	181 (80.4)	72 (78.3)	
Male	44 (19.6)	20 (21.7)	
**Patient Type, n (%)**			
Outpatient	181 (80.4)	80 (87.0)	0.196 ^a^
Inpatient	44 (19.6)	12 (13.0)	
**Setting, n (%)**			
**Outpatient Departments**			
General medicine	154 (85.1)	51 (63.7)	<0.001 ^b,^*
Obstetrics and gynecology	13 (7.2)	14 (17.5)	0.150 ^a^
Medicine	4 (2.2)	3 (3.8)	0.441 ^b^
Pediatrics	3 (1.7)	0 (0.0)	0.555 ^b^
Ear nose and throat	3 (1.7)	4 (5.0)	0.206 ^b^
Surgery	1 (0.6)	3 (3.8)	0.087 ^b^
Nephrology and dialysis	1 (0.6)	0 (0.0)	1.000 ^b^
Neurology	1 (0.6)	0 (0.0)	1.000 ^b^
Urology	1 (0.6)	2 (2.5)	0.223 ^b^
Oncology	0 (0.0)	3 (3.8)	0.028 ^b,^*
**Inpatient Departments**			
General medicine	26 (59.1)	3 (25.0)	0.052 ^a^
Obstetrics and gynecology	5 (11.4)	0 (0.0)	0.574 ^b^
Medicine	3 (6.8)	0 (0.0)	1.000 ^b^
Pediatrics	2 (4.5)	1 (8.3)	0.552 ^b^
Surgery	2 (4.5)	0 (0.0)	1.000 ^b^
Intensive care unit	2 (4.5)	6 (50.0)	0.001 ^b,^*
Nephrology and dialysis	1 (2.3)	0 (0.0)	1.000 ^b^
Neurology	1 (2.3)	0 (0.0)	1.000 ^b^
Oncology	1 (2.3)	2 (16.7)	0.113 ^b^
Cardiology	1 (2.3)	0 (0.0)	1.000 ^b^
**Specimen type, n (%)**			
Urine	178 (79.1)	73 (79.3)	1.000 ^a^
High vagina swab	13 (5.8)	6 (6.5)	1.000 ^a^
Stool	10 (4.4)	1 (1.1)	0.186 ^b^
Wound swab	10 (4.4)	1 (1.1)	0.186 ^b^
Ear swab	6 (2.7)	4 (4.3)	0.484 ^b^
Throat swab	3 (1.3)	1 (1.1)	1.000 ^b^
Sputum	2 (0.9)	1 (1.1)	1.000 ^b^
Catheter	2 (0.9)	2 (2.2)	0.583 ^b^
Semen	1 (0.4)	0 (0.0)	1.000 ^b^
Trachea aspirate	0 (0.0)	2 (2.2)	0.084 ^b^
Blood	0 (0.0)	1 (1.1)	0.290 ^b^

Abbreviation: IQR = Interquartile range. ^a^ Pearson chi-square test, ^b^ Fisher-exact test, ^c^ Independent *t*-test, * Statistically significant differences (*p* < 0.05).

**Table 3:** Prevalence of non-carbapenem-resistant and resistant isolates across bacterial species.

	Non-carbapenem Resistant Gram-Negative Bacilli (n = 225), n (%)	Carbapenem-Resistant Gram-Negative Bacilli (n = 92), n (%)
*Klebsiella pneumoniae*	101 (44.9)	26 (28.3)
*Escherichia coli*	84 (37.3)	43 (46.7)
*Pseudomonas aeruginosa*	10 (4.4)	13 (14.1)
*Enterobacter species*	10 (4.4)	0 (0.0)
*Proteus mirabilis*	9 (4.0)	6 (6.5)
*Enterobacter cloacae*	5 (2.2)	0 (0.0)
*Proteus* spp.	2 (0.9)	0 (0.0)
*Aeromonas fergusonii*	1 (0.4)	0 (0.0)
*Burkholderia cepacia*	1 (0.4)	0 (0.0)
*Raoultella ornithinolytica*	1 (0.4)	0 (0.0)
*Salmonella enterica*	1 (0.4)	0 (0.0)
*Acinetobacter baumannii*	0 (0.0)	2 (2.2)
*Enterobacter asburiae*	0 (0.0)	1 (1.1)
*Escherichia fergusonii*	0 (0.0)	1 (1.1)

**Table 4:** Antimicrobial resistance and carbapenemase gene profiles of presumptive carbapenemase-producing Gram-negative bacilli.

Sample Code	Organisms	Carbapenemase Gene	SAM	TS	TZP	CTX	CTZ	CAZ	CZA	FEP	IMP	MEM	CIP	GEN	AMK	COL	Classify
H1a	*P. aeruginosa*	*bla*_NDM_	-	-	R	R	R	R	R	R	R	S	R	R	S	I	XDR
R123	*P. aeruginosa*	Nd	-	-	R	R	R	R	R	R	R	S	R	R	S	S	XDR
L65	*P. aeruginosa*	Nd	-	-	R	R	R	R	R	R	R	R	S	R	R	I	XDR
L73	*P. aeruginosa*	Nd	-	-	R	R	S	R	R	R	R	S	R	S	S	S	MDR
L23	*P. aeruginosa*	Nd	-	-	R	R	R	R	R	R	R	S	S	R	S	R	XDR
L74	*K. pneumoniae*	*bla*_KPC_	R	R	R	R	R	R	R	R	R	R	R	S	I	S	XDR
L29	*K. pneumoniae*	*bla*_KPC_	R	R	R	R	R	S	S	S	I	I	S	S	-	I	MDR
L26	*K. pneumoniae*	*bla*_KPC_	R	R	R	R	R	S	S	S	I	I	S	S	-	I	MDR
R102	*K. pneumoniae*	*bla*_NDM_	R	R	R	R	R	R	R	R	R	R	R	R	I	S	XDR
L19	*E. coli*	*bla*_NDM_	R	R	R	R	R	R	R	R	R	R	R	R	R	R	PDR
R135	*E. coli*	*bla*_NDM_	R	R	R	R	R	R	R	R	R	R	R	R	R	S	XDR
H26	*E. coli*	*bla*_NDM_	R	R	R	R	R	R	R	R	R	R	R	R	S	S	XDR
R140	*E. coli*	Nd	R	R	R	R	R	R	R	R	R	R	R	R	S	S	XDR
R104	*A. baumannii*	*bla*_NDM_	R	R	R	R	R	R	R	R	R	R	R	R	S	S	XDR
R120	*A. baumannii*	*bla*_NDM_	R	R	R	R	R	R	R	R	R	R	R	S	I	S	XDR
R92	*E. fergusonii*	Nd	R	R	R	R	R	R	S	S	S	R	S	S	S	S	MDR
L61	*P. mirabilis*	Nd	R	R	S	S	S	S	S	S	R	S	S	I	S	S	MDR

Abbreviations: R, Resistance; I intermediate; S, Susceptible; SAM, Ampicillin/Sulbactam; TS, Trimethoprim/Sulfamethoxazole; TZP, Piperacillin/Tazobactam; CTX, Cefotaxime; CTZ, Ceftolozane/Tazobactam; CAZ, Ceftazidime; CZA, Ceftazidime/Avibactam; FEP, Cefepime; IMP, Imipenem; MEM, Meropenem; CIP, Ciprofloxacin; GEN, Gentamicin; AMK, Amikacin; COL, Colistin; MDR, Multidrug-resistance; XDR, Extensively drug-resistance; PDR, Pandrug-resistance; Nd, not detected; -, no analysis.

**Table 5:** Clinical and microbiological information on patients with carbapenem-resistant isolates.

Sample Code	Organisms	Hospital Unit/Department	Specimen	Diagnosis	30-Day Mortality Outcome
L23	*P. aeruginosa*	ICU	Wound Swab	Surgical wound infection	* No
L65	*P. aeruginosa*	ENT	Ear Swab	Otitis media	* No
L73	*P. aeruginosa*	ENT	Ear swab	Otitis media	* No
R123	*P. aeruginosa*	O&G	High Vaginal Swab	Pelvic Inflammatory Disease	* No
H1a	*P. aeruginosa*	Oncology	Throat Swab	Enlarged adenoid	* No
L19	*E. coli*	ICU	Blood	Benign neoplasia/Pituitary adenoma	** Died
H26	*E. coli*	O&G	Urine	UTI/Preterm premature rupture of membrane	* No
R135	*E. coli*	General Medicine/Surgery Outpatient	Urine	UTI	* No
R140	*E. coli*	General Medicine/Surgery Outpatient	Urine	UTI	* No
L26	*K. pneumoniae*	ICU	Catheter Tip	Prostate Enlargement/CVA	* No
L29	*K. pneumoniae*	ICU	Trachea Aspirate	Adenocarcinoma of the prostate/COPD	* No
L74	*K. pneumoniae*	General Medicine/Surgery Outpatient	Urine	UTI	* No
R10	*K. pneumoniae*	Pediatrics	Stool	Neonatal sepsis	* No
R104	*A. baumannii*	General Medicine/Surgery Outpatient	Urine	UTI	* No
R120	*A. baumannii*	General Medicine/Surgery Outpatient	Urine	Pyelonephritis/UTI	* No
R92	*E. fergusonii*	General Medicine/Surgery Outpatient	Urine	UTI	* No
L61	*P. mirabilis*	General Medicine/Surgery Outpatient	Urine	UTI	* No

Abbreviations: ICU, Intensive care unit; O&G, Obstetrics and gynecology; ENT, Ear nose and throat; UTI, Urinary Tract Infection; CVA, Cerebrovascular Accident; COPD, Chronic Obstructive Pulmonary Disease. * No mortality was recorded within 30 days. ** Patient died within 30 days.

## Data Availability

The data presented in this study are available on request from the corresponding author due to (specify the reason for the restriction).

## Supplementary Materials

The following supporting information can be downloaded at: https://www.mdpi.com/article/10.3390/idr17050119/s1, Table S1: Bacterial strains and their reference numbers used for quality control; Figure S1: Electrophoresis gel picture of *bla*_NDM_ gene; Figure S2: Electrophoresis gel picture of *bla*_KPC_ gene.

## References

[1] World Health Organization (2024). WHO Bacterial Priority Pathogens List, 2024: Bacterial Pathogens of Public Health Importance to Guide Research, Development and Strategies to Prevent and Control Antimicrobial Resistance.

[2] Hammoudi Halat D., Ayoub Moubareck C. (2020). The current burden of carbapenemases: Review of significant properties and dissemination among Gram-negative bacteria. Antibiotics.

[3] Pitout J.D.D., Peirano G., Kock M.M., Strydom K.A., Matsumura Y. (2019). The global ascendency of OXA-48-type carbapenemases. Clin. Microbiol. Rev..

[4] Olalekan A., Bader B.K., Iwalokun B., Wolf S., Lalremruata A., Dike A., Mannie-Udoh M., Lo Presti L., Liese J., Guther J. (2023). High incidence of carbapenemase-producing *Pseudomonas aeruginosa* clinical isolates from Lagos, Nigeria. JAC Antimicrob. Resist..

[5] Shaker O.A., Gomaa H.E., ElMasry S.A., Halim R.M.A., Abdelrahman A.H., Kamal J.S. (2018). Evaluation of Combined Use of Temocillin Disk and Mastdisks Inhibitor Combination Set Against Polymerase Chain Reaction for Detection of Carbapenem-Resistant Enterobacteriaceae. Open Access Maced. J. Med. Sci..

[6] Shettima S.A., Tickler I.A., Dela Cruz C.M., Tenover F.C. (2020). Characterisation of carbapenem-resistant Gram-negative organisms from clinical specimens in Yola, Nigeria. J. Glob. Antimicrob. Resist..

[7] Ghanbarinasab F., Haeili M., Ghanati S., Moghimi M. (2023). High prevalence of OXA-48-like and NDM carbapenemases among carbapenem resistant *Klebsiella pneumoniae* of clinical origin from Iran. Iran. J. Microbiol..

[8] Tula M.Y., Enabulele O.I., Ophori E.A., Aziegbemhin A.S., Iyoha O., Filgona J. (2023). A systematic review of the current status of carbapenem resistance in Nigeria: Its public health implication for national intervention. Niger. Postgrad. Med. J..

[9] Adesanya O.A., Igwe H.A. (2020). Carbapenem-resistant Enterobacteriaceae (CRE) and gram-negative bacterial infections in south-west Nigeria: A retrospective epidemiological surveillance study. AIMS Public Health.

[10] Ugah U.I., Udeani T.K. (2022). Prevalence of Phenotypic Carbapenem-Resistant Enterobacterales Isolates and Their Distribution by Sex, Age Groups, State and Species in South-East Nigeria. Gomal J. Med. Sci..

[11] Anibijuwon I.I., Gbala I.D., Adebisi O.O. (2018). Carbapenem-Resistant Enterobacteriaceae among In-Patients of Tertiary Hospitals in Southwest, Nigeria. Not. Sci. Biol..

[12] Reyes J., Komarow L., Chen L., Ge L., Hanson B.M., Cober E., Herc E., Alenazi T., Kaye K.S., Garcia-Diaz J. (2023). Global epidemiology and clinical outcomes of carbapenem-resistant Pseudomonas aeruginosa and associated carbapenemases (POP): A prospective cohort study. Lancet Microbe.

[13] Ling W., Furuya-Kanamori L., Ezure Y., Harris P.N.A., Paterson D.L. (2021). Adverse clinical outcomes associated with carbapenem-resistant *Acinetobacter* (CRA) infections: A systematic review and meta-analysis. JAC Antimicrob. Resist..

[14] Wilson G.M., Suda K.J., Fitzpatrick M.A., Bartle B., Pfeiffer C.D., Jones M., Rubin M.A., Perencevich E., Evans M., Evans C.T. (2021). Risk factors associated with carbapenemase-producing carbapenem-resistant Enterobacteriaceae positive cultures in a cohort of US Veterans. Clin. Infect. Dis..

[15] Kedisaletse M., Phumuzile D., Angela D., Andrew W., Mae N.F. (2023). Epidemiology, risk factors, and clinical outcomes of carbapenem-resistant Enterobacterales in Africa: A systematic review. J. Glob. Antimicrob. Resist..

[16] Odewale G., Adefioye O.J., Ojo J., Adewumi F.A., Olowe O.A. (2016). Multidrug resistance of *Acinetobacter baumannii* in Ladoke Akintola university teaching hospital, Osogbo, Nigeria. Eur. J. Microbiol. Immunol..

[17] Ogbolu D.O., Alli O.A.T., Oluremi A.S., Ogunjimi Y.T., Ojebode D.I., Dada V., Alaka O.O., Foster-Nyarko E., Webber M.A. (2020). Contribution of NDM and OXA-type carbapenemases to carbapenem resistance in clinical *Acinetobacter baumannii* from Nigeria. Infect. Dis..

[18] Health Facilities Monitoring and Accreditation Agency An Overview of Healthcare in Lagos. https://hefamaa.lagosstate.gov.ng/.

[19] Smith A., Hussey M. (2005). Gram Stain Protocols.

[20] European Committee for Antimicrobial Susceptibility Testing (2024). Breakpoint Table for Interpretation of MICs and Zone Diameters.

[21] Clinical and Laboratory Standards Institute (2023). Performance Standards for Antimicrobial Susceptibility Testing.

[22] Pasteran F., Lucero C., Soloaga R., Rapoport M., Corso A. (2011). Can we use imipenem and meropenem Vitek 2 MICs for detection of suspected KPC and other-carbapenemase producers among species of Enterobacteriaceae?. J. Clin. Microbiol..

[23] Koehne W.J., Peritz T., Privette K., Gould J. (2020). 1441. Using Carbapenem Resistance Levels to Discriminate Between Carbapenemase Producing and Non-Carbapenemase Producing Carbapenem Resistant Enterobacteriaceae. Open Forum Infect. Dis..

[24] Rafailidis P.I., Kofteridis D. (2022). Proposed amendments regarding the definitions of multidrug-resistant and extensively drug-resistant bacteria. Expert Rev. Anti Infect. Ther..

[25] Manchanda V., Rai S., Gupta S., Rautela R.S., Chopra R., Rawat D.S., Verma N., Singh N.P., Kaur I.R., Bhalla P. (2011). Development of TaqMan real-time polymerase chain reaction for the detection of the newly emerging form of carbapenem resistance gene in clinical isolates of *Escherichia coli*, *Klebsiella pneumoniae*, and *Acinetobacter baumannii*. Indian J. Med. Microbiol..

[26] Evans B.A., Amyes S.G. (2014). OXA beta-lactamases. Clin. Microbiol. Rev..

[27] Dallenne C., Da Costa A., Decre D., Favier C., Arlet G. (2010). Development of a set of multiplex PCR assays for the detection of genes encoding important beta-lactamases in Enterobacteriaceae. J. Antimicrob. Chemother..

[28] Nordmann P., Naas T., Poirel L. (2011). Global spread of Carbapenemase-producing Enterobacteriaceae. Emerg. Infect. Dis..

[29] Antimicrobial Resistance Collaborators (2022). Global burden of bacterial antimicrobial resistance in 2019: A systematic analysis. Lancet.

[30] van Duin D., Paterson D.L. (2016). Multidrug-Resistant Bacteria in the Community: Trends and Lessons Learned. Infect. Dis. Clin. N. Am..

[31] Li Q., Zhou X., Yang R., Shen X., Li G., Zhang C., Li P., Li S., Xie J., Yang Y. (2024). Carbapenem-resistant Gram-negative bacteria (CR-GNB) in ICUs: Resistance genes, therapeutics, and prevention—A comprehensive review. Front. Public Health.

[32] Mohammed M.U., Manisha D., Nagamani K. (2021). Clinical, phenotypic and genotypic profile of carbapenem resistant Gram negative infections in intensive care units. Indian J. Microbiol. Res..

[33] Ismail H., Zwane T.B.C., Du Toit E., da Costa R.M.A., Franceschi F., Perovic O. (2025). Carbapenem-resistant Enterobacterales among patients with bloodstream infections in South Africa: Consolidated surveillance data, 2015–2021. PLoS ONE.

[34] Maina J.W., Mutua J.M., Musyoki A.M. (2024). Carbapenem-resistant gram-negative bacterial infections and risk factors for acquisition in a Kenyan intensive care unit. BMC Infect. Dis..

[35] Venne D.M., Hartley D.M., Malchione M.D., Koch M., Britto A.Y., Goodman J.L. (2023). Review and analysis of the overlapping threats of carbapenem and polymyxin resistant *E. coli* and *Klebsiella* in Africa. Antimicrob. Resist. Infect. Control.

[36] Omoregbe F., Fagade O. (2020). Carbapenemase Producers among Gram Negative Bacteria from Environmental and Clinical Samples in Makurdi, Nigeria. J. Adv. Microbiol..

[37] Sabour S., Harrington K.R.V., Martinson E., Bhatnagar A.S., Huang J.Y., Duffy D., Bantle K., Lutgring J.D., Karlsson M., Brown A.C. (2024). Characterization of carbapenem-resistant Enterobacterales and Pseudomonas aeruginosa carrying multiple carbapenemase genes-Antimicrobial Resistance Laboratory Network, 2018–2022. J. Clin. Microbiol..

[38] Strich J.R., Lawandi A., Warner S., Demirkale C.Y., Sarzynski S., Babiker A., Dekker J.P., Kadri S.S. (2023). Association between piperacillin/tazobactam MIC and survival among hospitalized patients with Enterobacterales infections: Retrospective cohort analysis of electronic health records from 161 US hospitals. JAC Antimicrob. Resist..

[39] Karlowsky J.A., Lob S.H., Bauer K.A., Esterly J., Siddiqui F., Young K., Motyl M.R., Sahm D.F. (2024). Activity of ceftolozane/tazobactam, imipenem/relebactam and ceftazidime/avibactam against clinical Gram-negative isolates-SMART United States 2019–21. JAC Antimicrob. Resist..

[40] Leong Q., Chew K.L. (2022). Drug resistance rates of difficult to treat *Pseudomonas aeruginosa* isolates to ceftolozane-tazobactam and ceftazidime-avibactam from a tertiary hospital, Singapore. Pathology.

[41] Khan A.U., Maryam L., Zarrilli R. (2017). Structure, genetics and worldwide spread of New Delhi Metallo-beta-lactamase (NDM): A threat to public health. BMC Microbiol..

[42] Mohammed Y., Zailani S.B., Onipede A.O. (2015). Characterization of KPC, NDM and VIM type carbapenem resistance Enterobacteriaceae from North Eastern, Nigeria. J. Biosci. Med..

[43] Stoesser N., Phan H.T.T., Seale A.C., Aiken Z., Thomas S., Smith M., Wyllie D., George R., Sebra R., Mathers A.J. (2020). Genomic Epidemiology of Complex, Multispecies, Plasmid-Borne bla(KPC) Carbapenemase in Enterobacterales in the United Kingdom from 2009 to 2014. Antimicrob. Agents Chemother..

[44] Odih E.E., Oaikhena A.O., Underwood A., Hounmanou Y.M.G., Oduyebo O.O., Fadeyi A., Aboderin A.O., Ogunleye V.O., Argimon S., Akpunonu V.N. (2024). Correction for Odih et al., “High genetic diversity of carbapenem-resistant *Acinetobacter baumannii* isolates recovered in Nigerian hospitals in 2016 to 2020”. mSphere.

[45] Zhu S., Song C., Zhang J., Diao S., Heinrichs T.M., Martins F.S., Lv Z., Zhu Y., Yu M., Sy S.K.B. (2022). Effects of amikacin, polymyxin-B, and sulbactam combination on the pharmacodynamic indices of mutant selection against multi-drug resistant *Acinetobacter baumannii*. Front. Microbiol..

[46] Jean S.S., Harnod D., Hsueh P.R. (2022). Global threat of carbapenem-resistant Gram-negative bacteria. Front. Cell. Infect. Microbiol..

[47] Zhan Q., Xu Y., Wang B., Yu J., Shen X., Liu L., Cao X., Guo Y., Yu F. (2021). Distribution of fluoroquinolone resistance determinants in Carbapenem-resistant *Klebsiella pneumoniae* clinical isolates associated with bloodstream infections in China. BMC Microbiol..

[48] Vijayakumar S., Biswas I., Veeraraghavan B. (2019). Accurate identification of clinically important *Acinetobacter* spp.: An update. Future Sci. OA.

[49] Becker B., Weiss C., Holzapfel W.H. (2009). An evaluation of the use of three phenotypic test-systems for biochemical identification of Enterobacteriaceae and Pseudomonadaceae. Food Control.

[50] Jeong S., Hong J.S., Kim J.O., Kim K.H., Lee W., Bae I.K., Lee K., Jeong S.H. (2016). Identification of Acinetobacter Species Using Matrix-Assisted Laser Desorption Ionization-Time of Flight Mass Spectrometry. Ann. Lab. Med..

[51] MarÝ-Almirall M., Cosgaya C., Higgins P.G., Van Assche A., Telli M., Huys G., Lievens B., Seifert H., Dijkshoorn L., Roca I. (2017). MALDI-TOF/MS identification of species from the *Acinetobacter baumannii* (Ab) group revisited: Inclusion of the novel *A. seifertii* and *A. dijkshoorniae* species. Clin. Microbiol. Infect..

[52] De Florio L., Riva E., Giona A., Dedej E., Fogolari M., Cella E., Spoto S., Lai A., Zehender G., Ciccozzi M. (2018). MALDI-TOF MS identification and clustering applied to Enterobacter species in nosocomial setting. Front. Microbiol..

[53] van Duin D., Doi Y. (2017). The global epidemiology of carbapenemase-producing Enterobacteriaceae. Virulence.

[54] Pourhoseingholi M.A., Vahedi M., Rahimzadeh M. (2013). Sample size calculation in medical studies. Gastroenterol. Hepatol. Bed Bench.

