# Supplementary material for: Characterization of Carbapenem-Resistant Gram-Negative Bacilli Isolates in Multispecialty Private Hospitals in Lagos, Nigeria
Source: Infect Dis Rep. 2025 Sep 21;17(5):119. doi: 10.3390/idr17050119 (PMC12562451; doi:10.3390/idr17050119)
Supplement: Supplementary file 1 [file idr-17-00119-s001.zip › idr-3831956-supplementary.pdf]

Supplemental materials:

Characterization of Carbapenem-Resistant Gram-Negative Bacilli Isolates in Multispecialty Private Hospitals in Lagos, Nigeria

Table S1. Bacterial strains and their reference numbers used for quality control

Bacterial species	Reference number	Purpose
Pseudomonas aeruginosa	ATCC 27853	Quality control of Gram-staining and antimicrobial susceptibility testing
Escherichia coli	ATCC 25922	Quality control of Gram-staining and antimicrobial susceptibility testing
Escherichia coli	NCTC 13476	Positive control for bla_{IMP}
Klebsiella pneumoniae	NCTC 13438	Positive control for bla_{KPC}
Klebsiella pneumoniae	NCTC 13440	Positive control for bla_{VIM}
Klebsiella pneumoniae	NCTC 13443	Positive control for bla_{NDM}
Klebsiella pneumoniae	NCTC 13442	Positive control for bla_{OXA-48}

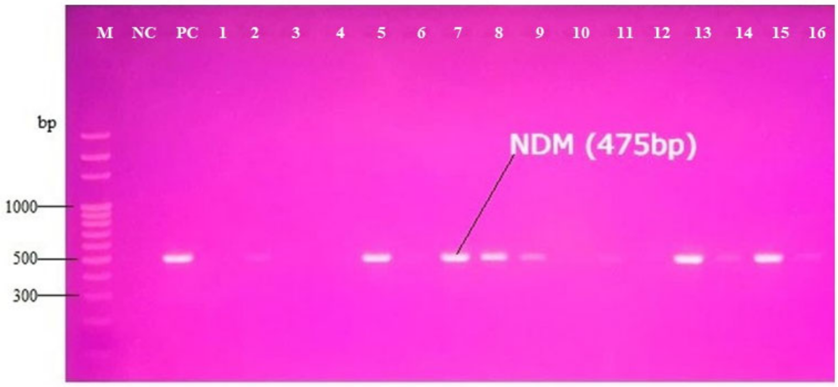

Figure S1. Electrophoresis gel picture of *bla*NDM gene. M = 100 bp ladder, NC = negative control, PC = *bla*NDM-positive control, and 5,7-9,13,15 = sample representative of *bla*NDM-positive isolates.

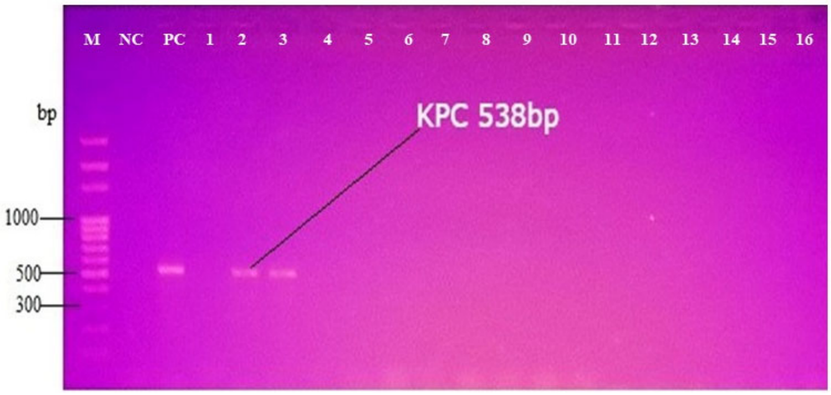

Figure S2. Electrophoresis gel picture of *bla*KPC gene. M = 100 bp ladder, NC = negative control, PC = *bla*KPC-positive control, and 2-3 = sample representative of *bla*KPC-positive isolates.
